# Event Recognition Based on Deep Learning in Chinese Texts

**DOI:** 10.1371/journal.pone.0160147

**Published:** 2016-08-08

**Authors:** Yajun Zhang, Zongtian Liu, Wen Zhou, National Natural Science Foundation of China (NSFC)

**Affiliations:** Shanghai University, School of Computer Engineering and Science, Shanghai, China; Centre national de la recherche scientifique, FRANCE

## Abstract

Event recognition is the most fundamental and critical task in event-based natural language processing systems. Existing event recognition methods based on rules and shallow neural networks have certain limitations. For example, extracting features using methods based on rules is difficult; methods based on shallow neural networks converge too quickly to a local minimum, resulting in low recognition precision. To address these problems, we propose the Chinese emergency event recognition model based on deep learning (CEERM). Firstly, we use a word segmentation system to segment sentences. According to event elements labeled in the CEC 2.0 corpus, we classify words into five categories: trigger words, participants, objects, time and location. Each word is vectorized according to the following six feature layers: part of speech, dependency grammar, length, location, distance between trigger word and core word and trigger word frequency. We obtain deep semantic features of words by training a feature vector set using a deep belief network (DBN), then analyze those features in order to identify trigger words by means of a back propagation neural network. Extensive testing shows that the CEERM achieves excellent recognition performance, with a maximum F-measure value of 85.17%. Moreover, we propose the dynamic-supervised DBN, which adds supervised fine-tuning to a restricted Boltzmann machine layer by monitoring its training performance. Test analysis reveals that the new DBN improves recognition performance and effectively controls the training time. Although the F-measure increases to 88.11%, the training time increases by only 25.35%.

## Introduction

Natural-language organized texts express higher-level semantic information through events. Recognizing those events can help computers easily understand the exact meaning of texts and lay a solid foundation for realizing event-based natural language processing (NLP) systems [1, 2]. We use trigger words to label events in texts. Typically, a trigger word is a major indicator of an event occurring and contains the maximum amount of information in a sentence. For example, in the sentence “Wenchuan suffered an earthquake on July 20, 2008.”, “earthquake” is a trigger word and therefore this event can be classified as an “earthquake” event. Its location element is “Wenchuan”. Differing from other elements that may be absent in an event, the trigger word is absolutely necessary for any event. Therefore, the essence of event recognition is trigger word recognition.

Current, widely-used trigger-word recognition methods are based on rules or shallow neural networks. However, these methods have certain limitations. For example, for methods based on rules, extracting features is difficult. They also have a poor portability. Methods based on shallow neural networks can not realize complex function approximations and suffer from the vanishing gradient problem, resulting in low recognition precision. In recent years, deep learning (DL) within the machine learning field [3] has shown that it can be successfully applied to reduce the data dimension by layer-wise training, abstract deep features of data and use those features to considerably improve classification precision. DL mimics the human brain’s information processing mechanism [4–6] in an unsupervised manner to learn a deep nonlinear network structure, achieves complex function approximations, obtains the feature functions of high-dimensional data and ultimately improves the accuracy of classification or prediction. DL has achieved breakthroughs in image identification, audio processing and video recognition. There are also preliminary applications of DL in many NLP fields such as syntactic analysis, entity relation extraction and emotional analysis. Glorot et al. [7] analyzed current recommendations and reviews on the internet, the number of which is exponentially increasing. As labeling these data is difficult, the researchers proposed a novel unsupervised study model based on DL which learns how to extract meaningful information from recommendation and review data on the internet. Xi et al. [8] proposed a Chinese anaphora resolution model based on deep belief networks (DBN). Although the recognition ability of the proposed method is not better than that of traditional support vector machine (SVM) anaphora resolution algorithms, the study provides a meaningful application of DL to Chinese NLP. Comparing to DL, Weng proposed a Finite Automaton in Developmental Network (FA-in-DN) [9], which incrementally learns the FA but takes sensory images directly and produces motor images directly.

Although DL can be applied to these fields on a preliminary basis [10, 11], there are few studies which show that the method can successfully extract deep semantic information in the field of event recognition. In this study, we propose a Chinese emergency event recognition model (CEERM) based on deep learning. We use a DL mechanism to mine deep semantic features automatically and analyze the role that these features play in event recognition. Using a feature analysis method, we abstract six feature layers of words in texts and then vectorize every word to a feature vector set. Finally, we analyze the feature vector sets using the CEERM to obtain recognition results. Test analysis shows that recognition performance in our model is better than that of existing methods.

Our model has some attractive features:

1. Based on a deep learning framework, it can approximate complex functions to obtain word semantics features effectively and contribute to classification.
2. Two types of classifiers are used in our model: unsupervised and dynamical supervised. One can alternate the classifier based on the application, which enhances the scalability and flexibility of the model.
3. It can overcome some of the shortcomings of exiting event recognition methods that are based on rules or shallow neural networks and can therefore improve recognition performance.
4. The model conducts a meaningful exploration for the application of DL to the NLP field and provides a foundation for its wider application in NLP.

### Related work

As a popular research field in NLP, event recognition technology has been widely applied to automatic summarization [12–15], automatic question answering [16–19] and information retrieval [20–23]. Existing event recognition methods are primarily divided into two types: those based on rules and those based on shallow neural networks. Methods based on rules commonly use predetermined rules to design templates for event recognition. For example, Yankova et al. [24] proposed a football event extraction system, and Lee et al. [25]developed a meteorological event extraction system based on ontology. Jiang [26]proposed a portable event pattern acquisition method which manually defines events, extracts tasks and automatically obtains a new event pattern from a raw corpus by building the mapping between event targets and corresponding event roles. Chen et al. [27] proposed an information extraction model based on event frames and built a catastrophic event frame which uses inheritance and inductive characteristics of the frames to simplify system implementation processes and summarize event information. Feng [28] proposed an emergency event information extraction method base on frames. It uses a predetermined event frame to extract news elements and examines new aspect information that was not pre-established in the frame as a complement to that same frame.

Methods based on shallow neural networks focus on constructing a classifier and the discovery, combination and selection of features. These methods consider event recognition as a classification problem and thus selecting appropriate features is critical for event classification. In 2002, Chieu and Ng introduced a maximum entropy classifier [29] and applied it for the purpose of recognizing events and their elements. For addressing the problem that existing event recognition methods did not consider context, Fu et al. proposed an event extraction method based on weight features of event elements in the event ontology [30]. In 2006, Ahn [31] proposed a method to recognize event classes and elements. In Ahn’s method, the most important step is identifying the most suitable trigger word to describe an event, then, use a classifier to recognize the class of this event, and lastly use the event class information to construct classifiers for each event element class to recognize them. Wang et al. [32] proposed a verb-driven method to extract event semantic information (5W1H) from Chinese texts. They proved its reliability and feasibility through extensive experimental data. Yankova et al. [24] proposed an event extraction method that integrates machine learning and semantic role labeling based on the PropBank corpus. This method achieved satisfactory results when applied to English language texts. McCracken et al. [33] extracted multiple elements using a syntax tree pattern matching method. Kim et al. [34] used a method that combines lexical semantics and semantic roles to recognize and classify events automatically.

Although current event recognition methods are efficient, certain limitations exist. Methods based on rules lack portability and robustness, have difficulties extracting features and need update rules to maintain optimal performance for new field texts. This requires many scientists with specialized knowledge, experienced linguists and time. Methods based on shallow neural networks search for optimal solutions by using a gradient descent algorithm. However, the nonlinear mapping between input and output of the algorithm converts the network error function space into a nonlinear one consisting of multiple minimal points. The gradient search direction is the only direction that reduces network errors; therefore, it always converges to a local minimum, with worsening performance as the number of network layers increases [35], critically affecting recognition performance. Considering the aforementioned problems, we propose the CEERM, which uses a DL model to obtain deep semantic features of event trigger words without manually designed rules and overcomes the local minimum problem. The model not only completes event recognition tasks, but also helps to recognize other event elements.

## Materials and Methods

### Study Model

#### Restricted Boltzmann machine(RBM)

**Fig 1:**
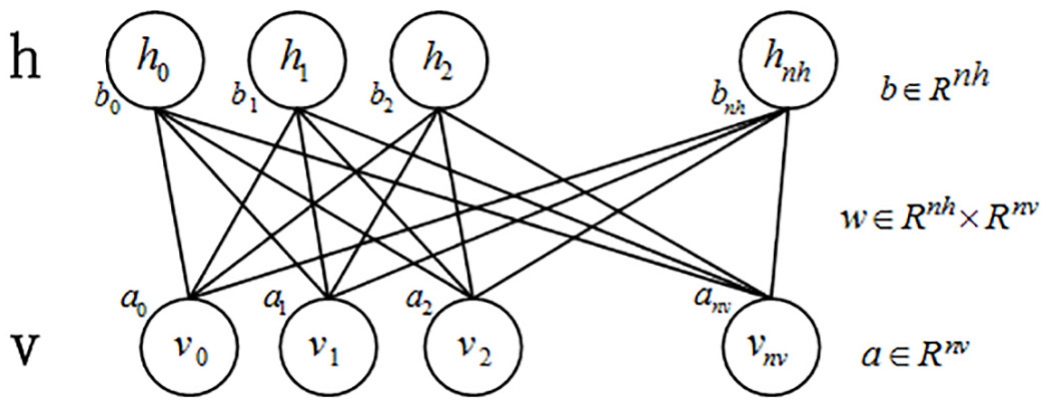
RBM structure.

RBM is a generative stochastic neural network proposed by Hinton and Sejnowsk [36] and is illustrated in Fig 1. This network consists of some visible units (mapping to visible variables that are data samples) and some hidden units (mapping to hidden variables). Visible or hidden variables are both binary variables whose state belongs to 0,1. The entire network is a bipartite graph and an edge connection exists only between visible and hidden units. This means that no edge connection exists within visible or hidden units. This represents the major difference between RBM and a general Boltzmann machine.

The energy function of the network structure is defined by Eq (1).

$$\begin{array}{cc} E_{\theta }\left(v,h\right)= & -\sum^{n_{v}}a_{i}v_{i}-\sum^{n_{h}}b_{j}h_{j}\\ & -\sum^{n_{v}}\sum^{n_{h}}h_{j}w_{j,i}v_{i} \end{array}$$ (1)

Where h and v represent hidden and visible layers, w represents the weight value between visible and hidden layers, and a and b represent their offset values, respectively. According to the known node value of a visible layer, we can obtain that of a hidden layer using the joint probability distribution equation [36] given by

$$P\left(h_{j}=1|v\right)=\frac{1}{1+exp(-b_{j}-\sum_{i=1}^{n_{v}}w_{j,i}v_{i})}$$ (2)

According to [36], RBM is a symmetric network. Therefore, we can obtain the node value of a visible layer from a known node value of a hidden layer using the equation as follows.

$$P\left(v_{i}=1|h\right)=\frac{1}{1+exp(-a_{i}-\sum_{j=1}^{n_{h}}w_{j,i}h_{j})}$$ (3)

The goal of training the RBM is to obtain the parameters *θ*(*w*, *a*, *b*), which maximize the joint probability p(v,h). The usual approach used to solve this problem is the Markov chain Monte Carlo(MCMC) algorithm. This approach constantly updates the node values of the hidden and visual layers until the Markov chain approach tends to be stable; thus, the joint probability p (v,h) reaches the maximum value [37]. The derivatives of the maximum and initial joint probability distributions are then obtained. Finally, the weight value *θ* is updated using the following equation, where *τ* is the iteration number and *η* is the learning speed.

$$\theta^{\left(\tau +1\right)}=\theta^{\left(\tau \right)}+\eta \frac{\partial \log P(v,h)}{\partial \theta }$$ (4)

Input vector *v*^0^ of the RBM is the vector of the visible layer when t = 0. Based on Eq (2), vector *h*^0^ of the hidden layer is obtained by *v*^0^ and *v*^1^ is the vector of the visible layer when time t = 1, obtained through *h*^0^ using Eq (3). In addition, *v*^∞^ and *h*^∞^ are the vectors of the visual and hidden layers, respectively, when time *t* = ∞. The slope of the joint probability distribution can be obtained by using the following equation.

$$\begin{array}{cc} \eta \frac{\partial \log P(v,h)}{\partial \theta } & =<h_{j}^{0}\left(v_{i}^{0}-v_{j}^{1}\right)>\\ & +<v_{i}^{1}\left(h_{j}^{0}-h_{j}^{0}\right)>+\cdots \\ & =<h_{j}^{0}v_{i}^{0}>-<h_{j}^{\infty }v_{i}^{\infty }> \end{array}$$ (5)

In Eq (5), *h*^0^*v*^0^ is the average value of point multiplication of the input feature vector and its corresponding feature vector in the hidden layer and *h*^∞^*v*^∞^ is the average value of the feature vector of the visible layer at the end of the Markov chain and its corresponding feature vector in the hidden layer. The Markov chain converges at *h*^∞^*v*^∞^. From Eq (5), the slope of the joint probability distribution is unrelated to the intermediate state and is concerned only with the initial and final states of the RMB network. According to Eq (4), the updated parameter *θ* can be obtained in order to implement self-training. In a traditional Markov chain approach, when solving for the maximum joint probability *P*(*h*^∞^*v*^∞^) and initial joint distribution probability p(v,h), guaranteeing the convergence rate is difficult. Moreover, the step ∞ is also difficult to calculate. Therefore, Hinton [38] proposed using contrastive divergence (CD) guidelines, which can speed up calculation without compromising accuracy. The Kullback-Leibler(KL) distance is used to measure the “difference” of two probability distributions, expressed as *KL*(*P*||*P*′), shown in the following equation.

$$CD_{n}=KL\left(P_{0}\right||P_{\infty })-KL\left(P_{n}\right|\left|P_{\infty }\right)$$ (6)

*CD*_*n*_ can be understood as a position measurement of *p*_*n*_ between *p*_0_ and *p*_∞_. Constantly assigning *p*_*n*_ to *p*_0_, we can obtain new values of *p*_0_ and *p*_*n*_ [39]. Experiments demonstrate that *CD*_*n*_ tends to 0 after r times of calculating the slope correction parameter, and the accuracy using the method is similar to that of the Markov chain approach. Therefore, this study uses the RBM network training method based on CD guidelines [40].

#### Deep belief network based on dynamic supervised learning

**Fig 2:**
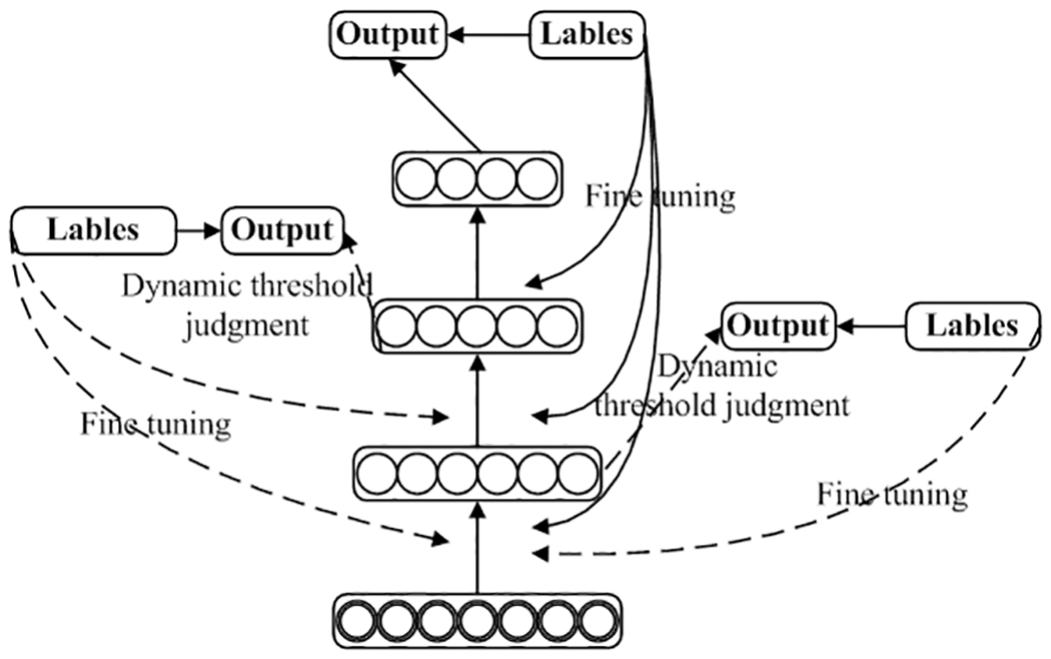
Structure of deep belief network based on dynamic supervision.

In the existing DBN network, the training way of each RBM layer is unsupervised; however, it is finally reverse supervised fine-tuned using the back propagation network (BP). The structure can improve training speed, but no supervision training exists between RBM layers. This can cause errors bottom-up propagation in RBM layers, which ultimately affects recognition performance. Therefore, we propose DBN based on dynamic supervision. In this network, RBM training results are estimated. Determining the use of BP training with supervision depends on the results, as shown in Fig 2. The average error and its standard deviation are used as evaluation criteria. If one of these is greater than the given threshold value, then the BP algorithm is used to fine-tune the RBM network parameters in order to perform training with supervision. Therefore, our DBN can reduce the risk of error propagating in RBM layers and thus improve recognition performance. Our method does not require supervised training in all RBM layers. Therefore, it limits DBN network training time and improves recognition performance.

Two evaluation parameters of DBN are used based on dynamic supervision: the average error of one time RBM training, as seen in Eq (7), and the unbiased standard deviation of errors of one time RBM training, as shown in Eq (8). We define the average value of total error and the average value of total error standard deviation as thresholds by training unsupervised DBN several times.

$$\bar{e}=\frac{e_{1}+e_{2}+e_{3}+\cdots +e_{n}}{n}=\frac{\sum_{i=1}^{n}e_{i}}{n}$$ (7)

$$S=\sqrt{\frac{\sum_{i=1}^{n}{\left(e_{i}-\bar{e}\right)}^{2}}{n}}$$ (8)

### Chinese emergency event recognition model based on deep learning(CEERM)

**Fig 3:**
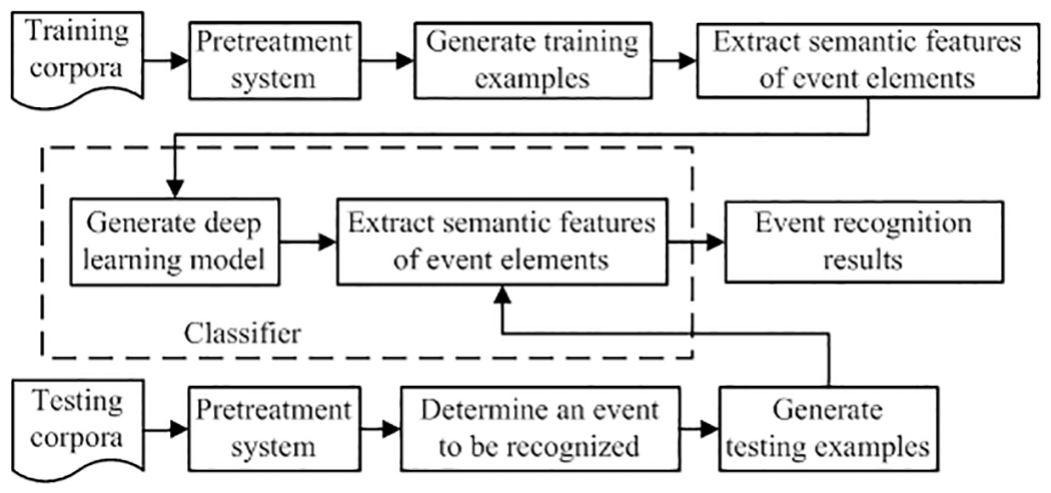
CEERM model frame.

CEERM has four main modules: corpus selection, a pre-processing system, feature vector generation and a deep classifier, as shown in Fig 3. The main function of the pre-processing module is to segment sentences in the corpus and then classify every word according to its labeled class. The feature vector generation module generates feature vectors for words based on six feature layers: part of speech layers (POSL), dependency grammar layers (DPL), length layers (LENL), location layers (LOCL), distance between trigger word and core word layers (DISL), and trigger words frequency layers (TFWL). Feature vectors are binary patterns. The deep classifier generates a stable DBN network by training the corpus and recognizing trigger words in the test corpus. We use two types of classifiers: without supervision and with dynamic supervision.

#### Corpus selection

Chinese Corpus CEC 2.0 (https://github.com/shijiebei2009/CEC-Corpus) is an event ontology corpus developed by the Semantic Intelligence Laboratory of Shanghai University. It has collected 333 newspaper texts about earthquakes, fires, traffic accidents, terrorist attacks and food poisoning. Descriptive statistics for these texts are shown in Table 1. We labeled event trigger words, participants, objects, time, location and other elements using a semi-automatic method. The corpus is divided into two parts: training and test. The training corpus contains 271 newspaper articles of five kinds obtained from CEC 2.0 and includes 51 articles about earthquakes, 61 about fires, 70 about traffic accidents, 50 about food poisoning, 39 about terrorist attacks, with a total of 4690 trigger words. The test corpus contains 1301 trigger words in 62 corpora of all five article types including 12 articles about earthquakes, 14 about fires, 15 about traffic accidents, 11 about food poisoning and 10 about terrorist attacks.

**Table 1:** Event Statistics in CEC 2.0.

Corpus Class	Section	Sentence	Event Sentence	Event	Ratio of Event Sentences(%)
Earthquake	63	496	463	1053	93.3
Fire	75	512	473	1216	92.4
Traffic accident	85	561	523	1790	93.2
Terrorist attacks	49	387	362	823	93.5
Food poisoning	61	439	409	1109	93.2
Total	333	2395	2230	5991	93.1

#### LTP segmentation system

The language technology platform (LTP) is a Chinese NLP system built by the Social Computing and Information Retrieval Research Center at the Harbin Institute of Technology. LTP has developed five Chinese NLP modules, including word segmentation, named entity recognition, morphology, sentence structure and semantics [41]. LTP has been widely recognized and praised, and the academic version of LTP has been shared with more than 500 research institutions free of charge.

#### Pre-processing system

**Fig 4:**
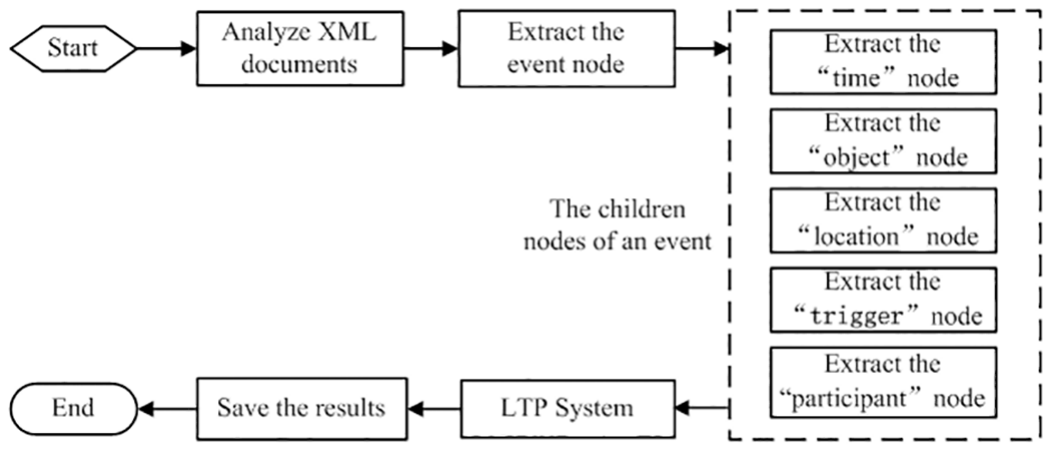
Flow of pre-processing system.

The main function of the pre-processing system is to process the XML format corpus files, obtain parts of speech (POS), a dependency grammar (DP) of words and finally to save the files in XML format. First, LTP segments the sentences into words. We then compare the words in this sentence with previously labeled trigger words. If the words are labeled trigger words, we classify them as trigger words. We classify other elements such as participants, objects, time and location using the same method. Pre-processing results form the foundation of feature representation and vector generation, detailed in Fig 4.

#### Deep classifier

The deep classifier uses word feature vectors as inputs and treats word classifications as training and test comparison criteria. The manner of training is such that it involves the training of the small batch pattern. Two kinds of classifiers are employed. One is an unsupervised DBN which consists of a multiple-layer unsupervised RBM and a single-layer supervised BP network. The other is based on dynamic supervised DBN and determines whether a supervised adjustment RBM network is required by analyzing the RBM training results. It thus controls training time and improves recognition performance.

#### Feature abstracting

Traditional event recognition methods based on features primarily use single learning mechanisms during the learning process. They do not consider the impact of feature layers on system learning; they only consider the role of the feature value. In fact, the human cognitive process has an abstract hierarchical nature. Recognizing unknown objects progresses from the abstract to the concrete. The higher the abstract level, the higher the recognition accuracy. Therefore, layering feature types and allowing them to perform their role adequately during the learning process is necessary.

According to the semantic features of trigger words in texts, we define six feature layers in order to transform the trigger word recognition into a feature classification problem. As DL can mine text features more effectively than traditional methods, it can recognize events with greater accuracy.

POSL: Part of speech is the most basic grammatical feature of a word. In a sentence, grammar imposes great restrictions on POS. For instance, nouns or pronouns can act as a subject, but using an adverb as the subject is absolutely impermissible. Therefore, taking POS as a feature layer conforms with the expression characteristics of text semantics information and the basic cognition when people identify events [42]. In CEC 2.0, trigger words have a centralized distribution, 80% of trigger words are verbs and 14% are nouns. Therefore, using POSL as a trigger word’s abstract feature layer can improve recognition accuracy.

DPL: According to dependency grammar, the predicate verb of a sentence is considered the center that dominates other sentence components. However, a predicate verb itself is not dominated by any other components and all dominated components subordinate to their dominator in one dependency relationship [43]. As dependency grammar exactly describes semantic role relationships between words, it has extremely high semantic performance. Containing the most information and having the clearest expression in a sentence, trigger words, in a certain sense, play the role as predicate verbs of dependency grammar. According to dependency grammar analysis of trigger words in CEC 2.0, 62% of their role in dependency grammar is head and 18% is verb-object. In most cases, the predicate verbs of dependency grammar accord with trigger words.

LENL: In any sentence, a close connection exists between a word’s length (LEN) and its role [44]. For instance, the subject and object usually are longer than the predicate because the predicate more often expresses action. In addition, words of this kind are usually more concise and provide clearer expression. Length analysis of trigger words in CEC 2.0 shows that the length of 81% of trigger words is 2 and of 12% of words is 1. Due to its centralized distribution, this feature is crucial in recognizing trigger words effectively.

LOCL: The word index in a sentence denotes a word’s position, which affects its semantic information. Word position in a sentence is a kind of specification that takes form gradually during the writing process. Every language places words having different roles in different positions based on the language’s unique grammatical logic. Therefore, a word index has a positive meaning for determining the role a word plays in a sentence [45]. Considering that sentence lengths are different, we use only 11 location indices (0 to 10) to annotate the abstract layer. Location (LOC) analysis in CEC 2.0 indicates that 16% of trigger words are in the first position of sentences and 12% are in the second position. The location feature contributes minimally to recognition accuracy because of its low distribution concentration degree.

DISL: Core words are the main components of sentences, which means they usually express the core content of the sentence. Trigger words express the most important content of a sentence [46]. Therefore, trigger words and core words have a high degree of similarity. The shorter the distance (DIS) between a regular word and a core word, the greater is the possibility that the former is a trigger word. In this study, a core word refers to a word whose dependency grammar role is head. Analyzing CEC 2.0, we find that the distance between trigger and core words in 12% of the cases is 0 and in 27% the distance is 1.

TWFL: Many trigger words reappear in CEC 2.0. Some trigger words appear more often than others. Trigger word frequency (TWF) is divided into eight grades: hp, a, b, c, d, e, f, and g, definitions of which are as given in Table 2.

**Table 2:** Grade rules of trigger word frequency.

Identifier	Feature abstract layer	Grade	Define
101	TWFL	hp	Fr ≥ 1
102	TWFL	a	Fr> 1 and Fr≤ 5
103	TWFL	b	Fr> 5 and Fr≤ 10
104	TWFL	c	Fr> 10 and Fr≤ 20
105	TWFL	d	Fr> 20 and Fr≤ 30
106	TWFL	e	Fr> 30 and Fr≤ 40
107	TWFL	f	Fr> 40 and Fr≤ 50
108	TWFL	g	Fr> 50

Note: Fr denotes Frequency.

#### Feature representation and samples generation

Feature representation mainly uses a binary representation model. Feature vector generation of TWFL is accumulated and five other vector generations of abstract layers follow the same pattern. We describe all feature representations in detail below.

Feature representation of POSL: Twenty-nine dimensions of POS feature vectors represent 29 types of POS. If the POS of a candidate word corresponds with that of a certain dimension representation of a feature vector, then the feature vector value of the word at the dimension is 1, whereas that at the remaining 28 dimensions are 0.

Feature representation of DPL: On this feature layer, 14 vector dimensions exist, thus representing 14 kinds of DP.

Feature representation of LENL: On this layer, the vector dimension is 10, meaning the candidate word length ranges from 1 to10. If a word length exceeds 10, then every feature vector value of the candidate word is 0.

Feature representation of LOCL: The vector dimension is 11, denoting 11 index positions from 0 to 10. If an index position exceeds 10, then every feature vector value of the candidate word is 0.

Feature representation of DISL: The vector dimension is 11, representing DIS from 0 to 10. If it is greater than 10, then each feature vector value of the candidate word is 0.

Feature representation of TWFL: The dimension is 8, representing 8 grades of a trigger word. This layer’s vector representation uses the accumulation approach. For example, the grade of the candidate word “earthquakes” is e. Therefore, the feature vector value is “1 1 1 1 1 1 0 0”.

Based on the aforementioned feature representations, we give the following example: “The police arrested the suspect on the spot last night.”. In this sentence, Types 1, 2, 3, and 4 represents the participant, time, trigger word and location, respectively. The results are shown in Table 3.

**Table 3:** Example of feature representation.

Candidate word	Feature abstraction layer	Feature vector value	Class
last night	POSL	00000000000000000100000000000	2
	DPL+LENL	00000010000000 0100000000	
	LOCL+DISL+TWFL	10000000000 00010000000 00000000	
police	POSL	00000000000100000000000000000	1
	DPL+LENL	10000000000000 0100000000	
	LOCL+DISL+TWFL	01000000000 00100000000 00000000	
on the spot	POSL	00010000000000000000000000000	4
	DPL+LENL	00000010000000 0100000000	
	LOCL+DISL+TWFL	00100000000 01000000000 00000000	
arrested	POSL	00000000000000000000000010000	3
	DPL+LENL	00000000000001 0100000000	
	LOCL+DISL+TWFL	00010000000 10000000000 11000000	
suspect	POSL	00000000000100000000000000000	1
	DPL+LENL	01000000000000 0010000000	
	LOCL+DISL+TWFL	00001000000 01000000000 00000000	

## Results

We used the CEC 2.0 Chinese corpus for experimental data and then extracted semantic information required for event recognition according to the feature representations described in the Feature abstracting section. Then, based on the process described in the Feature representation and samples generation section, training and test samples are generated. We rapidly trained the training samples using CEERM to generate a stable deep classifier, which could recognize the events in test samples. The values of deep-classifier-related parameters numepochs, batchsize, momentum, and alpha are set to 1, 100, 0, and 1, respectively. In our experiments, we used precision (P), recall rate (R), and F-measure to evaluate recognition performance. In addition, we used F incremental (FI), F incremental rate (FIR), F comparison incremental (FCI), and F comparison incremental rate (FCIR) to contrast the recognition performance of the two classifiers.

### Threshold analysis of dynamic supervised classifier

The dynamic supervised classifier monitors the average and standard deviation of training errors in a single RBM training period. If either exceeds a threshold, the classifier uses the BP network to fine-tune the RBM layer to achieve the following: ensure error is controlled within a specific range, reduce the probability of error being propagated layer-wise and improve recognition performance. However, the choice of the threshold impacts recognition performance and training time. If the threshold is too low, fine-tuning during each RBM training period is possible, which greatly improves model training time. If the threshold is too high, it may reduce the possibility of eliminating those errors in the lower layer in time and may thus propagate to the upper layer, resulting in low recognition performance. In this study, we use the average and standard deviation of errors as the threshold values after several rounds training the unsupervised DBN. In Table 4, we increase the amount of test data to obtain test results. In these tests, the number of RBM layers is four, all feature layers are added and RBM1 represents the error in the first RBM layer training. Analysis of the parameters in Table 4 enables us to set the dynamic classifier threshold to an average of 4 and the standard deviation to 2.

**Table 4:** Threshold analysis of dynamic supervised classifier.

Model+Abstract Layers	Number of vectors	RBM1(%)	RBM2(%)	RBM3(%)	RBM4(%)	Average(%)	Standard deviation(%)
DBN4+ POSL+DPL+ LENL+LOCL+ DISL+TWFL
	2000	7.25	4.17	2.61	2.52	4.14	2.21
	4000	7.18	4.09	2.52	2.49	4.07	2.2
	6000	7.13	4.09	2.51	2.48	4.05	2.19
	8000	7.12	4.11	2.52	2.45	4.05	2.19
	10000	7.08	4.01	2.46	2.42	3.99	2.19
	12000	7.01	3.99	2.45	2.46	3.98	2.15
	14000	6.98	3.97	2.42	2.41	3.95	2.15
	16000	6.97	3.93	2.38	2.41	3.92	2.16
	18000	6.9	3.89	2.35	2.36	3.88	2.14
	20000	6.86	3.91	2.38	2.32	3.87	2.13
Average value	11000	7.048	4.016	2.46	2.432	3.99	2.17

### CEERM recognition performance of unsupervised classifier

We combine the features of POS, DP, LEN, LOC, DIS and TWF to generate training and testing samples, then use DBNi to complete two sets of experiments. Thereafter, we compare them. I represents the number of RBM layers contained in DBN with the values set to 1,2,3,4,5,6,7 and 8, respectively. In the first experiment, we test recognition performance with the same feature layers while the number of RBM layer increases. In the second experiment, we test the performance when introducing different semantic feature layers with the same RBM layers.

Table 5 shows the results of the first experiment. An increase in the number of RBM layers can improve recognition performance when the number is below five. The values of DBN4 performance indices R, P and F are 87.32%, 83.12% and 85.17%, which are highest in all results. Compared to the result of DBN1, FI and FIR increase by 5.73% and 7.21%. As the model can extract the main feature information from data using multiple-layer mapping units, its effect is greater than that of a single-layer structure. However, when the number of RBM layers is greater than four, its recognition performance degrades with each increase in the number of RBM layers. The values of DBN8 result indices R, P and F are only 82.59%, 80.12% and 81.34%. This means that the recognition performance is not directly proportional to the increase in the number of RBM layers. Therefore, in an actual application, we should choose a suitable depth of RBM based on extensive testing and analysis. In addition, system training time increases as the number of RBM layers increases, because the number of neural network training nodes increases and therefore the workload of combining and training increases dramatically. This establishes a higher demand for the processing ability of the system platform.

**Table 5:** Unsupervised CEERM recognition performance based on different numbers of RBM layers.

Model+Abstract Layers	R	P	F	FI	FIR(%)
DBN1+POSL+DPL+LENL+LOCL+DISL+TWFL	80.4	78.5	79.44	0	0
DBN2+POSL+DPL+LENL+LOCL+DISL+TWFL	83.2	78.7	80.89	1.45	1.82
DBN3+POSL+DPL+LENL+LOCL+DISL+TWFL	84.7	82.8	83.74	2.85	3.53
DBN4+POSL+DPL+LENL+LOCL+DISL+TWFL	87.32	83.12	85.17	1.43	1.71
DBN5+POSL+DPL+LENL+LOCL+DISL+TWFL	86.13	83.21	84.64	−0.52	−0.61
DBN6+POSL+DPL+LENL+LOCL+DISL+TWFL	85.21	81.21	83.16	−1.48	−1.75
DBN7+POSL+DPL+LENL+LOCL+DISL+TWFL	83.13	81.32	82.22	−0.95	−1.14
DBN8+POSL+DPL+LENL+LOCL+DISL+TWFL	82.59	80.12	81.34	−0.88	−1.07

Note: *F*_*n*_ is the F measure when the number of RBM layers is n, FI is difference between *F*_*n*_ and *F*_*n*−1_,

FIR is equal to $\frac{F_{n}-F_{n-1}}{F_{n-1}}$.

The second experiment tests the recognition performance of the CREEM model when introducing different feature layers. The results, shown in Table 6, reveal that introducing different feature layers can considerably improve overall performance. With an increase in the number of feature layers, all three performance indices improve. However, different feature layers contribute differently to the improvement in recognition performance. From Table 6, we see that DPL contributes most to performance, with FI and FIR increasing by 5.62% and 7.83%, because the DP can precisely describe semantic role relationships. Using relationship information can therefore add the performance when recognizing events. LENL also has a big contribution to the performance improvement, with FI and FIR increase by 3.16% and 4.08%. The reason for this improvement is that many verbs and verb noun phrases are used as trigger words. These phrases are mainly composed of two or three single words so the model will easily identify such trigger words through length. LOCL contributes minimally, FI and FIR only increase by 0.52% and 0.65%, because event sentences don’t have a consistent length. Furthermore, even if two event sentences have a approximate length, the location of their trigger words may not be consistent leading to a smaller overall contribution. DISL contributes little to recognition performance, with FI and FIR increase by 2.01% and 2.48%, because the written style is irregular for Chinese and the distance between a core word and a trigger word becomes more uncertain. If we use a corpus of another language with a clear writing style as our experimental data, the DISL may contribute more. Due to the test corpus and training corpus being disjoint, the TWFL only retains the frequency of trigger words in the training corpus. This reduces the contribution to the performance of the model.

**Table 6:** Unsupervised CEERM recognition performance based on different feature layers.

Model+Abstract Layers	R	P	F	FI	FIR(%)
DBN4+POSL	78.13	66.32	71.74	0	0
DBN4+POSL+DPL	92.42	66.52	77.36	5.62	7.83
DBN4+POSL+DPL+LENL	83.87	77.42	80.52	3.16	4.08
DBN4+POSL+DPL+LENL+LOCL	83.87	78.39	81.04	0.52	0.65
DBN4+POSL+DPL+LENL+LOCL+DISL	80.01	86.32	83.05	2.01	2.48
DBN4+POSL+DPL+LENL+LOCL+DISL+TWFL	87.32	83.12	85.17	2.12	2.56

Note: *F*_*c*_ is the F measure when the number of feature layers is c, FI is difference between *F*_*c*_ and *F*_*c*−1_,

FIR is equal to $\frac{F_{c}-F_{c-1}}{F_{c-1}}$.

### CEERM recognition effect comparison of two classifiers

To compare our two classifiers, we test recognition performance as the number of DBN and feature layers increase. We also compare and analyze the stability of the classifiers with respect to recognition performance as the number of DBN layers increases to a limited degree. We then compare their training time.

A comparison of the unsupervised and dynamic supervised classifiers is shown in Table 7. The average increment of the three indices and the total absolute increment of the dynamic supervised classifier are greater than that of the unsupervised classifier when the numbers of RBM layers increases. The highest FCI and FCIR are 5.56% and 6.43%. As the structure of dynamic supervised DBN can hinder the errors propagating layer by layer, recognition performance is increasing. At the same time, we also show that the FI and FIR of the two DBN decline when the numbers of RBM layers is more than four, but that the rate of decline is small for dynamic supervised DBN and therefore has a higher stability. We can conclude that adding dynamic supervised learning can improve recognition performance to a certain extent.

**Table 7:** Comparison of recognition effect between dynamic supervised and unsupervised CEERM based on different numbers of RBM layers.

Model+Abstract Layers	R	P	F	FI	FIR(%)	FCI	FCIR(%)
DBN1+POSL+DPL+LENL+LOCL+DISL+TWFL	80.4	78.5	79.44	0	0	0	0
DBN2+POSL+DPL+LENL+LOCL+DISL+TWFL	89.5	83.6	86.45	7.01	8.83	5.56	6.43
DBN3+POSL+DPL+LENL+LOCL+DISL+TWFL	89.6	84.7	87.08	0.63	0.73	3.34	3.84
DBN4+POSL+DPL+LENL+LOCL+DISL+TWFL	90.32	86	88.11	1.03	1.18	2.94	3.34
DBN5+POSL+DPL+LENL+LOCL+DISL+TWFL	90.3	85	87.57	−0.54	−0.61	2.93	3.35
DBN6+POSL+DPL+LENL+LOCL+DISL+TWFL	89.7	84.8	87.18	−0.39	−0.44	4.02	4.61
DBN7+POSL+DPL+LENL+LOCL+DISL+TWFL	89.1	84.2	86.58	−0.6	−0.69	4.36	5.04
DBN8+POSL+DPL+LENL+LOCL+DISL+TWFL	90.1	82.4	86.08	−0.5	−0.58	4.74	5.51

Note: *F*_*d*_ is the F measure of the dynamic supervised classifier, *F*_*u*_ is the F measure of the unsupervised classifier, FCI is difference between *F*_*d*_ and *F*_*u*_, FCIR is equal to $\frac{F_{d}-F_{u}}{F_{u}}$.

Table 8 shows a comparison of the unsupervised and dynamic supervised classifiers. The experimental results show that an increasing number of feature layers can considerably improve overall performance for the two classifiers. DPL contributes the most whereas LOCL contributes minimally. Each layer’s contribution is the same as that of the unsupervised classifier. The effects of feature layers in event recognition are very similar for the two classifiers. However, the dynamic supervised classifier has better performance than unsupervised, the highest FCI and FCIR are 4.89% and 6.83%, the minimal are 1.7% and 2.0%, and the average are 2.67% and 3.26%. In conclusion, the recognition performance of the dynamic classifier is better than that of the unsupervised classifier, which means the way of supervision is very important for the performance of deep learning, and we should treat it as a key factor when using deep learning in NLP applications.

**Table 8:** Comparison of recognition effect between dynamic supervised and unsupervised CEERM based on different feature layers.

Model+Abstract Layers	R	P	F	FI	FIR(%)	FCI	FCIR(%)
DBN4+POSL	75.11	78.21	76.63	0	0	4.89	6.38
DBN4+POSL+DPL	79.13	80.35	79.74	3.11	4.05	2.38	2.98
DBN4+POSL+DPL+LENL	83.21	80.81	81.99	2.26	2.83	1.47	1.79
DBN4+POSL+DPL+LENL+LOCL	87.32	78.61	82.74	0.74	0.91	1.7	2.05
DBN4+POSL+DPL+LENL+LOCL+DISL	91.76	80.28	85.64	2.9	3.51	2.59	3.02
DBN4+POSL+DPL+LENL+LOCL+DISL+TWFL	90.32	86	88.11	2.47	2.88	2.94	3.34

Note: See the note to Table 7.

**Fig 5:**
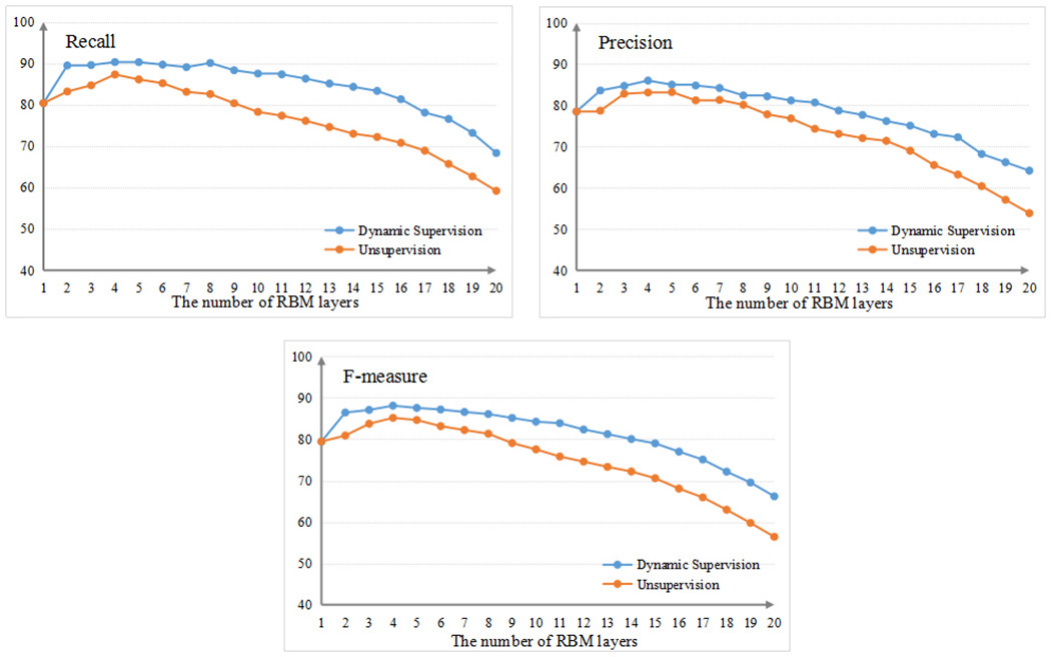
Comparison of the stability of the two classifiers.

Fig 5 illustrates our comparisons and analysis of the stability of the classifiers regarding recognition performance. We analyzed their stability by increasing the number of RBM layers in the same number of feature layers. Fig 5 shows that with an increase in RBM layers, errors at the bottom layer propagate upward to higher layers and the recognition performance of the unsupervised classifier declines gradually, the difference between the maximum and minimum of the three indexes R, P, F are 28.11%, 29.25% and 28.75%. In addition, the classifier has a large amplitude and poor stability. By contrast, the dynamic supervised classifier has high stability, the difference between the maximum and minimum of the three indexes R, P, F are 21.97%, 21.83% and 21.91%, which are all lower than the values of the unsupervised classifier.

Table 9 shows the training time of the two deep classifiers. The table shows that the training time of the dynamic supervised classifier is longer because adding fine-tuning increases the training time. However, the increase is less than 30%. Considering the improved performance, we can allow such a limited increase in time. The classifier without supervision has a shorter training time. We chose the classifier’s way of supervision according to specific situations and platform performance in actual applications. If training efficiency is emphasized, the classifier without supervision is the better of the two classifiers. Dynamic supervision is chosen because of its better recognition performance.

**Table 9:** Comparison of the training time of the two classifiers.

Test condition	Training time of one period	U(ms)	D(ms)	Increment Rate(%)
All feature layer are the same Increasing the RBM layers from 1 to 8	Average time	1290	1595	23.64
	Total time	9761	12235	25.35
RBM layer is fixed Increasing the feature layers from 1 to 6	Average time	1089	1317	20.94
	Total time	6135	7574	23.46

Note: U refers to the unsupervised classifier and D refers to the dynamic supervised classifier.

We compared the recognition performance of CEERM based on rules and SVM. Table 10 shows the experimental results. We can conclude from these results that the DL-based method has the best recognition performance. They also reveal that DL is extremely effective in text mining and suitable for a wider application in NLP.

**Table 10:** Recognition performance of CEERM, based on rules and SVM.

Methods	Supervision way	R	P	F
CEERM	unsupervision	87.32	83.12	85.17
CEERM	dynamic supervision	90.32	86	88.11
Based on rules	--	80.3	78.6	79.44
Based on SVM	--	84.5	81.1	82.77

Although the method proposed in this paper can overcome the limitations of the existing event recognition methods and offers a great improvement in the recognition performance and stability, the analysis of our experimental results reveals that the classification performance does not reach 100%. This is likely due to a number of different reasons;

1. DBN is an effective multi-classification deep neural network and the recognition performance of DBN is generally better than that of shallow layer neural networks. However, regardless of whether the application is to image recognition or other areas, the method has not been able to achieve a 100% classification result. This is why the method proposed in this paper also fails to achieve a perfect recognition result.
2. In this paper, the testing and training corpus are disjoint, leading to the semantic features of some events in the test corpus not being learned by the deep classifiers, which can affect recognition performance. But if the testing and training corpus are joint, then the scalability of the model will be affected to a certain extent. Especially during application, it may be necessary to process a large number of non-labeled texts. This scenario requires that the testing and training corpus are disjoint.
3. This method is mainly used for event recognition in Chinese texts. Different from English NLP, Chinese NLP must use word segmentation technology. In this paper, we apply the LTP of the Harbin Institute of Technology as a segmentation tool, which has a higher recall and precision rate, but has at the same time still failed to reach 100% recognition performance. The errors due to an imperfect application of LTP also influence the model recognition performance negatively, thereby reducing the overall model recognition performance.
4. In order to improve event recognition performance, this paper proposes a variety of event recognition features. However these do not capture all features of events, there may be other potential features to be excavated, which may improve recognition performance of the system. In future research, we believe that we need to mine more features so as to achieve the best possible recognition results.

## Discussion

In this study, we examined event recognition problems combining semantic information. We transformed event recognition into semantic feature classification and proposed CEERM by applying DL to event recognition. Our method performed better than existing event recognition methods. Based on the DL framework, CEERM can obtain deep features of text data to improve event recognition performance. We also designed two kinds of classifiers. One is the unsupervised classifier, which is efficient but shows poor recognition performance. The other is the dynamic supervised classifier, which not only improves recognition performance but also limits training time. Our study shows that the dynamic supervised classifier obtains the most comprehensive results.

We also tested the recognition performance of CEERM. Experimental results show that recognition performance can be improved when the number of RBM training layers increases to a certain extent. Simultaneously, the relationship between recognition performance and increasing number of RBM layers is nonlinear. When the RBM layers reach a certain number, the recognition performance of the two classifiers diminishes. Therefore, we must choose suitable RBM layers based on specific application requirements. Introducing feature layers to CEERM can help improve recognition performance. In fact, test results show that adding feature layers can improve system performance regardless of the number of RBM layers in CEERM.

In the future, the performance and scalability of CEERM can be improved. In addition, this model could be applied to other element recognition in event ontology. Although DL has achieved breakthroughs in audio and image recognition, it has not yet a considerable influence on NLP. Thus, the connections between words, sentences and paragraphs are relatively obscure. A limited RBM structure of an existing DL can not express the vector structure of texts precisely. Therefore, designing a DL model that is suitable for text analysis may become a trend in future research.
